# Using Machine Learning to Study Factors Affecting Discharge Destination in Recovery Units

**DOI:** 10.7759/cureus.70916

**Published:** 2024-10-06

**Authors:** Kenta Kunoh, Hiroki Bizen, Keisuke Fujii, Daiki Nakashima, Daisuke Kimura

**Affiliations:** 1 Department of Rehabilitation, Yamada Hospital, Gifu, JPN; 2 Department of Occupational Therapy, Kansai University of Health Sciences, Osaka, JPN; 3 Department of Rehabilitation Occupational Therapy, Suzuka University of Medical Science, Suzuka, JPN; 4 Department of Rehabilitation, Naragakuen University, Nara, JPN; 5 Department of Occupational Therapy, Nagoya Women’s University, Aichi, JPN

**Keywords:** discharge destination, machine learning, random forest, recovery units, stroke

## Abstract

Background: In recent years, machine learning has been developed in the medical community to construct multidimensional datasets consisting of many variables and perform simultaneous factor analysis.

Objective: This study aimed to construct a multidimensional dataset of 50 items by incorporating supervised machine learning in a random forest algorithm to predict whether patients will be discharged home or to a facility after a stroke.

Methods: Thirty patients hospitalized with cerebrovascular diseases who were subsequently discharged were considered as the study subjects. The dataset used for analysis consisted of attributes such as characteristics (three items), physical and cognitive functions (seven items), functional independence measure (FIM) (18 items), blood data (16 items), and social characteristics (six items). The discharge destination variable was either a home or a facility. Machine learning was used to extract factors important for this classification. The accuracy of the random forest was calculated by five-fold cross-validation. The mean decrease Gini, a measure of importance in classification, was calculated for each fold.

Results: The results indicated that FIM, a measure of activities of daily living (ADL), and cognitive function, including memory, which strongly influenced the prediction equation, were important factors in the proposed algorithm. The results of the analysis revealed that the algorithm predicted home discharge or institutionalization with 87.1% accuracy.

Conclusion: Through this study, ADL and cognitive function were identified as important factors in predicting home discharge for patients with cerebrovascular disease.

## Introduction

Stroke patients face physical, cognitive, emotional, and social challenges following their illness. To address these challenges, rehabilitation is practiced during hospitalization. During the rehabilitation period, patients can enjoy professional support in the hospital environment and can be independent in activities of daily living (ADL) in the hospital, but higher ADL abilities are needed to live independently at home after discharge. In deciding where to discharge stroke patients, home and facility are very different, and the impact on post-discharge life is immeasurable. Therefore, it is often difficult to decide where stroke patients should be discharged from the hospital because the patient's own wish to "go home despite the aftereffects" conflicts with the family's wish to "live safely in a facility." So, which is better for stroke patients: home or a facility? Previous studies have indicated that many stroke patients have the ultimate goal of rehabilitation to return home [1]. In addition to this, it has been reported that returning home after a stroke with sequelae can maintain a better quality of life (QOL) than living in an institution [2]. In light of these previous studies, it can be said that therapists need to help stroke patients return home as much as possible, as long as the patients want to do so, a fact that is widely known in the medical field.

However, at present, the return-to-home rate for patients discharged from hospitals specializing in rehabilitation remains roughly 60％, leaving important issues to be addressed in helping stroke patients return home. On the other hand, in order for stroke patients to return home, it is common to examine the most influential factors among the many problems that arise and prioritize rehabilitation interventions. Therefore, it is extremely important to know the factors that influence the return of stroke patients to home, and this information is necessary to prioritize the problems. Prior studies have examined many factors that influence the return of stroke patients to home, and it is widely recognized that ADLs are among the most influential [3]. Other physical functional, laboratory, and environmental aspects have also been noted to have an impact [4-10]. However, the results obtained are not uniform, since many of the previous studies used relatively narrowly defined items within a certain range as explanatory variables, and each report used different explanatory variables, despite the fact that a variety of factors are noted to affect the return to home. In order to solve such problems, in recent years, machine learning that constructs multidimensional datasets consisting of many variables and performs factor analysis simultaneously has been developed. Machine learning is a technology that gives data to a computer and has it learn patterns and regularities from that data. Using this makes it possible to automatically classify and predict things. Furthermore, when using conventional multivariate analysis, a large number of subjects is required depending on the variables, but by using machine learning, it is possible to obtain stable results even with a relatively small number of subjects. In fact, previous studies using machine learning have been conducted on neuroimaging of dementia and on the classification of subtypes of cognitive profiles of autism spectrum disorders, and it can be said that the development of machine learning is remarkable even in the medical community [11,12]. Therefore, in order to solve the problems that have arisen so far and to clarify the true factors that affect the return of patients with cerebrovascular disorders to their homes, it can be said that the use of machine learning, which is constructed from multidimensional data sets and performs factor analysis, is necessary. The ability to construct an algorithm that accurately predicts the factors that affect the return of patients with cerebrovascular disorders to their homes will lead to the determination of intervention priorities in rehabilitation settings.

Random forest is one of the analytical methods used to predict the return of cerebrovascular accident patients to their homes from multidimensional data sets. Random Forest is a statistical method that predicts classification from large data sets and has several features. One is that it is highly accurate, and because it is a method that combines multiple decision trees, it is generally possible to achieve a high level of accuracy. Second, it suppresses overfitting, and because it constructs multiple decision trees using random subsets, it has features that make it less prone to overfitting than individual decision trees. Even with a small sample, it is possible to avoid reflecting the characteristics of the group and to assume the trend of the population.

Therefore, in this study, we constructed a multidimensional dataset consisting of 50 data points, and used Random Forest, a supervised machine learning method, to predict whether a stroke patient would be discharged home or to a facility using random forest.

## Materials and methods

The study participants were 30 stroke patients who were hospitalized in a convalescent rehabilitation ward from January 2023 to June 2023, and then discharged to their homes or facilities. The 30 participants consisted of 19 male and 11 female patients with a mean age of 71.5±13.4 years. Previous studies have reported that the following factors affect the discharge of stroke patients from hospital to home: length of stay, whether or not they live with family, physical function, cognitive function, liver function, kidney function, nutritional status, anemia, ability to perform ADL, number of falls during hospitalization, recurrence, and medical history [10,13-24]. Therefore, we created a total of 50 items based on the following: characteristics (three items), physical and cognitive functions (seven items), functional independence measure (FIM) that indicates the degree of independence in daily life (18 items), blood data (16 items), and social characteristics (six items). The evaluation parameters are listed in Table 1. The discharge destination was dichotomized according to whether the patient was discharged to a home or a facility.

**Table 1:** List of evaluation data

	Evaluation data
Characteristic	Age
	Sex
	Requiring nursing care
Physical and cognitive functions	Revised version of Hasegawa's Dementia Scale (HDS-R)
	Knee extensor strength (paralyzed side/non-paralyzed side)
	Grip strength (paralyzed side/non-paralyzed side)
	Berg Balance Scale
	6-min walk test
Functional independence measure (FIM)	Eating
	Dressing-upper
	Dressing-lower
	Grooming
	Bathing
	Toileting
	Sphincter control bladder
	Sphincter control bowel
	Transfer (chair)
	Transfer (toilet)
	Transfer (tub)
	Locomotion walk/wheelchair
	Locomotion stairs
	Communication comprehension
	Communication expression
	Social interaction
	Problem-solving
	Memory
Blood data	Red blood cell (RBC)
	Hematocrit (Ht)
	Hemoglobin content (Hb)
	Platelet (PLT)
	Mean corpuscular hemoglobin concentration (MCHC)
	Mean corpuscular volume (MCV)
	Mean corpuscular hemoglobinconcentration (MCH)
	White blood cell (WBC)
	Albumin (Alb)
	Total protein (TP)
	Alanine aminotransferase (ALT)
	Aspartate aminotransferase (AST)
	C-reactive protein (CRP)
	Blood urea nitrogen (BUN)
	Creatinine (Cre)
Social characteristic	Days in hospital
	Number of falls
	Place of discharge
	Recurrent stroke
	Past medical history
	Revision of fall assessment and classification of risk
	Living together

In the analysis using machine learning, a random forest was conducted with the presence or absence of returning home as a dependent variable and all other variables as independent variables; an algorithm that can classify the presence or absence of returning home was created, and the factors important for classification were extracted. In the random forest, all independent variables underwent five-fold cross-validation using training data, and each fold was randomly divided into training and test data. The training data were fed into a random forest to create an algorithm that classified whether the patient would be discharged home or to an institution, and the test data were fed into the obtained algorithm to predict the classification. The accuracy of the training and test data was calculated for each fold, and the average of the five folds was calculated. The mean decrease Gini, a measure of importance in classification, was calculated for each fold, and the average of five folds was calculated thereafter.

The “RandomForest” package of R version 4.3.0 was used for data analysis. Data were collected retrospectively from electronic medical records of the relevant items. The study was conducted in accordance with the Declaration of Helsinki and the data obtained were anonymized so that personal information could not be identified. In conducting this study, the subjects were given a written explanation of the study and signed a consent form. The Research Ethics Review Committee of Kansai University of Health Sciences approved the study (approval number: 22-16) before the experiments were conducted.

## Results

Table 2 shows the descriptive statistics used to indicate the attributes of the subjects.

**Table 2:** Descriptive statistics for all evaluation items

	Evaluation data	Home	Facility
Characteristic	Age	71.5±14.4	71.7±11.0
	Sex (men/women)	13/8	6/4
	Requiring nursing care	2.2±2.2	3.9±2.7
Physical and cognitive functions	Revised version of Hasegawa's Dementia Scale (HDS-R)	25.0±2.2	15.8±6.7
	Knee extensor strength (non-paralyzed side)	42.0±16.8	37.4±17.9
	Knee extensor strength (paralyzed side)	32.3±11.7	23.9±16.7
	Grip strength (non-paralyzed side)	26.4±12.4	21.9±13.2
	Grip strength (paralyzed side)	19.3±13.2	12.3±10.3
	Berg Balance Scale	47.4±9.2	31.0±18.3
	6-min walk test	315.0±143.9	187.7±167.0
Functional independence measure (FIM)	Eating	6.5±0.5	5.5±0.8
	Dressing-upper	6.4±1.5	3.6±2.2
	Dressing-lower	6.4±1.6	3.5±2.3
	Grooming	6.6±0.8	5.3±0.9
	Bathing	5.9±1.9	3.7±2.0
	Toileting	6.4±0.8	3.6±2.5
	Sphincter control bladder	6.4±0.8	3.7±2.5
	Sphincter control bowel	6.5±0.6	3.8±2.4
	Transfer (chair)	6.4±0.8	4.8±1.2
	Transfer (toilet)	6.4±0.8	4.6±1.6
	Transfer (tub)	5.2±1.8	3.0±2.5
	Locomotion walk/wheelchair	6.1±1.4	3.1±2.5
	Locomotion stairs	5.0±1.5	3.7±2.4
	Communication comprehension	6.2±1.3	4.5±1.7
	Communication expression	6.0±1.3	4.8±1.5
	Social interaction	6.7±0.8	5.3±1.3
	Problem solving	6.2±1.4	3.2±2.3
	Memory	5.9±1.8	2.9±1.9
Blood data	Red blood cell (RBC)	429.2±53.6	422.7±41.7
	Hematocrit (Ht)	39.7±4.5	39.3±3.4
	Hemoglobin content (Hb)	13.3±1.8	14.2±4.2
	Platelet (PLT)	21.5±5.8	25.5±9.1
	Mean corpuscular hemoglobin concentration (MCHC)	33.1±0.8	32.5±1.2
	Mean corpuscular volume (MCV)	92.8±3.7	92.9±3.2
	Mean corpuscular hemoglobinconcentration (MCH)	30.6±1.2	30.2±1.6
	White blood cell (WBC)	5879.0±1420.2	6226.0±1331.9
	Albumin (Alb)	4.0±0.4	3.7±0.5
	Total protein (TP)	6.7±0.5	6.3±0.7
	Alanine aminotransferase (ALT)	27.3±30.5	20.4±12.4
	Aspartate aminotransferase (AST)	23.8±13.9	20.2±5.9
	C-reactive protein (CRP)	0.3±0.4	0.2±0.3
	Blood urea nitrogen (BUN)	14.6±3.7	17.8±8.9
	Creatinine (Cre)	0.8±0.2	0.9±0.3
Social characteristic	Days in hospital	63.8±31.2	77.1±36.9
	Number of falls	0.3±0.5	0.2±0.3
	Place of discharge	19	11
	Recurrent stroke (have/not have)	0/19	0/11
	Past medical history (have/not have)	11/8	8/2
	Revision of fall assessment and classification of risk	1.8±0.7	2.5±0.8
	Living together	1.5±1.0	1.8±1.2

**Figure 1:**
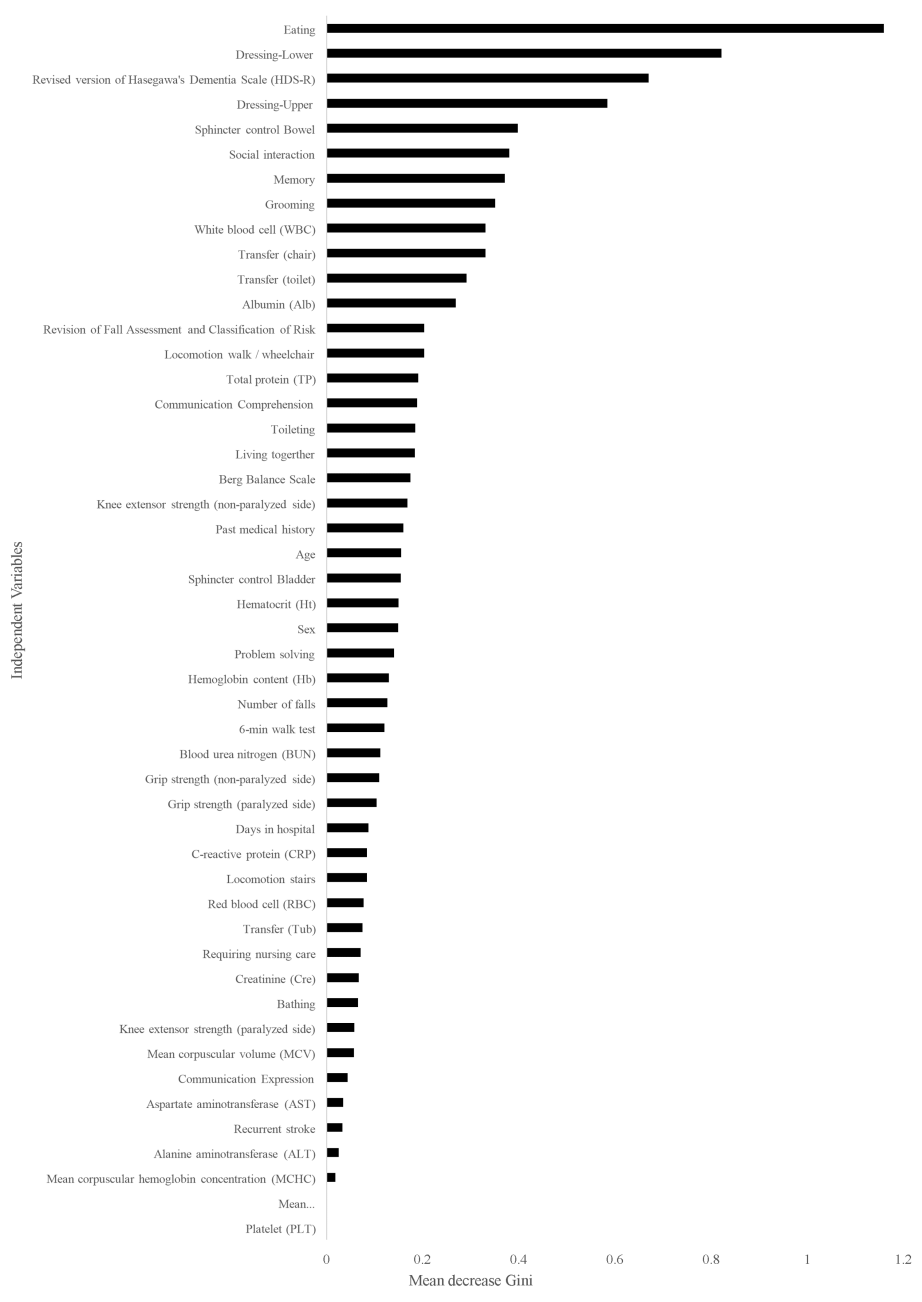
Mean decrease Gini of 49 items

In addition, the results of the random forest showed that the average five-fold accuracy of the training data was 100% and that of the test data was 87.1%. The top seven mean decrease Gini were FIM eating, FIM dressing-lower, FIM dressing-upper, HDS-R, social interaction, sphincter control bowel, and memory. The mean decrease Gini was 1.0933 for FIM eating, 0.8139 for FIM dressing-lower, 0.5993 for FIM dressing-upper, 0.5993 for HDS-R, 0.5971 for social interaction, 0.4220 for sphincter control bowel, and 0.3707 for memory. The top seven items showing the mean decrease Gini included six ADLs and one cognitive function (Figure 1).

## Discussion

In this study, we constructed an algorithm for predicting the discharge destinations of hospitalized patients through machine learning using random forests. The results showed that the algorithm predicted either home discharge or institutionalization with an accuracy of 87.1%. In this study, an accuracy of 87.1% was recorded for 30 subjects, which is said to be an extremely good result. This is because a low accuracy indicates over-learning. In other words, it can be said that random forest shows the possibility of performing multivariate analysis using many variables with a small number of subjects compared to conventional statistical methods. The key items of the algorithm were the FIM, a measure of ADL, and cognitive function, including memory, which strongly influenced the prediction equation.

Previous studies have reported the relationship between ADLs and discharge destinations. For example, previous studies have reported that the degree of independence in ADL and medical necessity are important predictors of where a patient is discharged [25-27]. In addition, previous studies have reported that these ADLs are important factors in returning home, not only for motor function but also for cognitive function [28]. In this study too, many ADLs, including not only motor functions but also cognitive functions, were cited as important factors.

Regarding these points, first, the reason why meals were listed as an important factor is that meals in FIM indicate whether or not a person is able to feed themselves and whether or not they are able to eat orally, and in ADL, meals are positioned as a movement with a low level of difficulty [29,30]. In other words, whether or not a person can perform low-level eating activities can be interpreted as a screening assessment of whether or not they can perform other ADL activities. From the above, it is thought that this will have a significant impact on returning to one's home. Next, when we check previous research on the reasons why dressing-lower and dressing-upper were cited as important factors, it is mentioned that dressing behavior is important for returning home [31]. It has also been reported that patients who can perform dressing-upper independently have a lower rate of motor impairment than those who cannot [32]. Furthermore, many stroke patients are undergoing rehabilitation approaches that focus on dressing and undressing at a high frequency in order to learn new dressing and undressing movements [33]. In other words, if the ability to dress oneself declines after a stroke, there is a high possibility that motor impairment will be observed, and high-frequency rehabilitation intervention will be necessary in order to acquire new dressing skills. If the patient is unable to learn how to change their clothes, the caregiver will have to help them every day. Specifically, because they are required to use a method of care that takes into account pain and falls, it is assumed that the burden of care will increase, and this is thought to be a factor in determining whether they can return home.

Next, we will look at the reasons why social interaction was cited as an influencing factor in returning to work from home. Social interaction refers to whether you are able to interact appropriately with others. In particular, when it comes to rehabilitation situations, it can be said that things like refusing rehabilitation, verbal violence, and neglect are not conducive to positive rehabilitation [29]. The fact that it is difficult to connect to active rehabilitation in this way leads to a situation where differences in the amount of rehabilitation, the frequency of rehabilitation, and the amount of load are likely to occur, and it is suggested that this has an indirect effect on returning home.

It was also found that the degree of independence in sphincter control of the bowel affects the ability to return home. Previous studies have also shown that the presence or absence of fecal incontinence affects the ability to return home [34,35]. As to the reason for this, it has been reported that recognizing fecal incontinence increases the sense of burden of caregivers of stroke patients [36]. This sense of burden from caring for someone is thought to lead to physical and mental fatigue, financial strain, and confusion, and to situations where people are forced to choose to move into a facility in order to avoid caring for someone.

Next, we will look at why cognitive function was cited as an important factor. It is widely known that developing dementia makes it difficult to return home [37,38]. In this study, we can say that the same trend was shown in previous studies. However, in this study, memory in FIM was also listed as a factor that affects returning home. A review of the research shows that there is a deep connection between memory impairment and dementia after a stroke [39]. FIM memory is scored based on recognition of people with whom you have a close relationship in your daily life, recognition of your daily routine, and fulfillment of requests from others. Therefore, the recognition of cognitive or memory decline is interpreted as requiring assistance with daily routines and tasks, and the caregiver must constantly prompt the person with dementia to perform actions. However, in reality, there are not many situations where you can always stay by the patient's side, and this can be a factor that leads to them choosing to enter a facility.

In addition to this study, a multidimensional dataset consisting of 50 variables, including five major items, was constructed and used for analysis. In supervised machine learning, the accumulation of variables ensures the accuracy of the algorithm. Therefore, when machine learning is used to predict a dependent variable, the construction of a dataset consisting of more than a certain number of items would be important.

Among these, previous studies have reported the importance of including cognitive function in the data set [38]. Some studies have reported that it is difficult to accurately predict where stroke patients will be discharged using a single cognitive function data set, but it has been reported that adding physical function to cognitive function improves the accuracy of predicting where stroke patients will be discharged [40,41]. In this study, we also predicted where patients would be discharged to, including ADL items and cognitive function, and as a result, the prediction accuracy was high, supporting the results of previous studies. Therefore, we believe that the cognitive function dataset can improve the accuracy of predicting the discharge destination by adding to one of the several items, rather than using it alone.

Due to the limitations of the small number of subjects in this study, future studies should include more subjects, for example by conducting multicenter studies. Furthermore, as this is a retrospective study, the results need to be interpreted carefully while taking these factors into account. In addition, although we used activity level and physical and mental function as variables in this study, it is thought that social background and other factors may also affect the return to home, so it is thought that including these as variables may improve the accuracy of the results. We think that addressing these issues is a challenge.

## Conclusions

This study constructed an algorithm for predicting the discharge destinations of hospitalized patients through machine learning using random forests. The algorithm predicted either home discharge or institutionalization with an accuracy of 87.1%. The algorithm showed that the ability to perform ADLs and cognitive function is important for patients with stroke to return home. Additionally, constructing a dataset from a large number of endpoints was suggested to increase the accuracy of prediction for returning home.
